# Hypercoagulable Rotational Thromboelastometry During Hospital Stay Is Associated with Post-Discharge DLco Impairment in Patients with COVID-19-Related Pneumonia

**DOI:** 10.3390/v16121916

**Published:** 2024-12-14

**Authors:** Natasa-Eleni Loutsidi, Marianna Politou, Vassilios Vlahakos, Dimitrios Korakakis, Theodora Kassi, Asimina Nika, Abraham Pouliakis, Konstantinos Eleftheriou, Evangelos Balis, Apostolos G. Pappas, Ioannis Kalomenidis

**Affiliations:** 1 Haematology-Lymphomas Department and Bone Marrow Transplant Unit, “Evangelismos” General Hospital, 10676 Athens, Greece; 2 Hematology Laboratory-Blood Bank, Aretaieion Hospital, School of Medicine, National and Kapodistrian University of Athens, 11528 Athens, Greece; mariannapolitou@gmail.com (M.P.); dimitris_korakakis@yahoo.gr (D.K.); theokassi@gmail.com (T.K.); asiminanik2@gmail.com (A.N.); 3 1st Department of Critical Care and Pulmonary Medicine, School of Medicine, National and Kapodistrian University of Athens, “Evangelismos” General Hospital, 10676 Athens, Greece; vvlahakos@hotmail.com (V.V.); coselef@hotmail.com (K.E.); apo.pappas88@gmail.com (A.G.P.); ikalom@med.uoa.gr (I.K.); 4 2nd Department of Pathology, National and Kapodistrian University of Athens, “ATTIKON” University Hospital, 12462 Chaidari, Greece; apou1967@gmail.com; 5 Department of Pulmonary Medicine, “Evangelismos” Hospital, 10676 Athens, Greece; evanbalis@yahoo.co.uk

**Keywords:** ROTEM, COVID-19, pneumonia, long-COVID, DLco, thromboelastometry

## Abstract

Hypercoagulation is central to the pathogenesis of acute and post-acute COVID-19. This prospective observational study explored whether rotational thromboelastometry (ROTEM), a method that unveils coagulation status, predicts outcomes of hospitalized patients with COVID-19 pneumonia. We investigated 62 patients using ROTEM that was conducted at enrollment, clinical deterioration, discharge and follow-up visits 1 and 3 months post-discharge. A hypercoagulable ROTEM was more common at clinical deterioration than at enrollment and the levels of hypercoagulable ROTEM indices correlated with the clinical severity score. Hypercoagulable ROTEM at enrollment was not associated with in-hospital death. Patients with hypercoagulable ROTEM at enrollment, discharge and 1 month post-discharge had an increased risk of persistent symptoms 1 and 3 months after discharge. Patients with hypercoagulable ROTEM at enrollment, discharge, and 1 month after discharge were more likely to have lung diffusion capacity (DLco) impairment 3 months after discharge. High levels of hypercoagulable ROTEM indices were associated with the increased risk of persistent symptoms at later stages of the disease. In a multivariate analysis, (i) hypercoagulable ROTEM at discharge and female gender were linked to the presence of symptoms at one month post-discharge, (ii) hypercoagulable ROTEM at one month after discharge was linked to the presence of symptoms at three months post-discharge, (iii) hypercoagulable ROTEM at enrollment and at discharge and female gender were linked to the presence of impaired DLco at three months post-discharge. Excessive coagulation may contribute to long-COVID pathogenesis and ROTEM findings during hospitalization may predict post-acute-COVID-19 sequelae in patients with COVID-19-related pneumonia.

## 1. Introduction

COVID-19 pneumonia, is characterized by excessive coagulation activity and thrombotic complications including large vessel thrombosis and thrombosis in the microcirculation, which can lead to organ dysfunction, including Adult Respiratory Distress Syndrome (ARDS), and may persist during the period following acute infection [1,2]. Furthermore, there are considerable data indicating that endothelial dysfunction and associated microvascular thrombosis are involved in the pathogenesis of the post-acute-COVID-19 syndrome [3,4,5,6]. In patients with COVID-19, standard coagulation tests, such as the International Normalized Ratio (INR), D-Dimers, fibrinogen levels, prothrombin time (PT), activated partial thromboplastin time (aPTT) and platelet number are not sensitive enough for detecting hypercoagulability [7]. On the other hand, rotational thromboelastometry (ROTEM), a global coagulation test, evaluates clot formation and lysis in vitro and can provide a detailed view of coagulation/hemostasis system activity [8]. ROTEM assesses the dynamics of thrombin, fibrinogen level and activity and fibrinolysis [9].

In the present study, we investigated hospitalized patients with COVID-19-related pneumonia using sequential ROTEM analysis at different clinically relevant time points, during and after the hospital stay. We hypothesized the following: (a) hypercoagulability at the time of enrollment, as determined by ROTEM, predicts clinical deterioration and fatal outcome of the acute disease; (b) at clinical deterioration, patients are more likely to present a hypercoagulable ROTEM profile; (c) clinical severity is associated with hypercoagulable ROTEM parameters; (d) a hypercoagulable ROTEM profile during hospital stay predicts the persistence of symptoms and abnormal lung diffusion capacity after discharge.

## 2. Methods

### 2.1. Study Design

We prospectively enrolled non-ICU patients with COVID-19-related pneumonia hospitalized at the “Evangelismos” Hospital between April 2021 and March 2022. The study was conducted according to the principles of the Declaration of Helsinki and its amendments and was approved by the Ethics Committee of “Evangelismos” General Hospital (Athens, Greece), protocol number: 136-29/04/2021. All participants signed an informed consent form.

The inclusion criteria were as follows: a positive nasopharyngeal swab real-time quantitative reverse transcription PCR (qRT-PCR) test for SARS-CoV-2 and hospitalization because of COVID-19 pneumonia. The exclusion criteria were dementia, pregnancy, age < 18 years old, active malignant disease, active thromboembolic event, recent surgery (within 3 months), participation at a clinical trial, and the hesitation or inability to attend the outpatient clinic of our hospital after discharge. Patients received treatment and respiratory support according to the national guidelines for COVID-19. After discharge, patients were followed at the outpatient COVID-19 clinic following a typical schedule of visits at 1 and 3 months after discharge. The follow-up included clinical, imaging and pulmonary functional [spirometry and lung diffusion capacity (DLco)] tests conducted at 3 months post-discharge evaluation. Spirometry and lung diffusion capacity were assessed using Jaeger MasterScreen PFT, Carefusion, Hoechberg, Germany.

The primary outcome of the study was “hospital mortality”. Secondary outcomes included “clinical deterioration during hospital stay”, the “presence of symptoms at 1 and 3 months post-discharge” and “DLco at 3 months post-discharge”.

ROTEM, conventional coagulation tests [D-Dimers, activated partial thromboplastin time (aPTT), international normalized ratio (INR)] and assessments of platelet counts and mean platelet volume (MPV) were carried out at the following time points: at the time of study enrollment, at the time of clinical deterioration, at discharge, 1 month after their discharge, and 3 months after discharge, only for those who had a thrombotic ROTEM 1 month after their discharge. Their lipid profile was also recorded on admission, since hyper-cholesterolaemia and hyper-triglyceridaemia are recognised risk factors for vascular complications. In addition, treatment with nonsteroidal anti-inflammatory drugs (NSAIDs), E-EPA, daily use of fish oil, or antihemorrhagic drugs on admission, during hospital stay and up to 3 months post discharge was recorded.

ROTEM deterioration was defined as the conversion from a normal to a thrombotic or hemorrhagic profile at consecutive measurements. Clinical deterioration was defined as an increasing score of the WHO clinical progression scale (CPS) [10].

### 2.2. ROTEM Analysis

ROTEM analysis was conducted in the Hematology Laboratory-Blood Bank, Aretaieio Hospital. Native ROTEM (NATEM) and NATEM with the addition of heparinase (NaHEPTEM) were used. The addition of heparinase degrades the heparin that is present in the sample and therefore allows for the ROTEM analysis in heparinised samples. A total of 5 mL of whole blood with no additional reagents was processed immediately after collection. The samples were placed into a fixed cuvette and a cylindrical spike was inserted into the blood and rotated around its longitudinal axis. When the blood clot appeared, the rotation was restricted and clot firmness was raised. A signal was generated and transferred from the pin into an integrated computer to demonstrate the ROTEM graph and its parameters [11,12]. Apixaban does not affect ROTEM parameters [13]. The following ROTEM parameters were derived: CT (clotting time, sec), CFT (clot formation time, sec), MCF (maximum clot firmness, sec), A10 and A20 [Amplitudes (mm) at 10 and 20 min, respectively], Alp (α-angle, degrees), LI30% and LI60% (the percentage of thrombus lysis in 30 and 60 min, respectively). The normal ranges of the above mentioned ROTEM parameters are depicted in Supplementary Table S1. ROTEM results were further characterized as hypercoagulable and hemorrhagic [14] as described in Supplementary Table S1.

### 2.3. Statistical Analysis

The statistical analysis was performed using the SAS for Windows, Version number 9.4 software platform (SAS Institute Inc., Cary, NC, USA) (DiMaggio, 2013; SAS Institute, 2014) and Graphpad Prism Software (Version 9, San Diego, CA, USA). Descriptive values were expressed as the mean (standard deviation), for normally distributed data, and median [inter-quartile range (IQR)], when data were found not to be normally distributed. Comparisons between groups for the qualitative parameters were made using the chi-square test (and if required a Fisher exact test was performed). For the continuous parameters (if normality was ensured), we applied parametric tests, such as the *t*-test or ANOVA; otherwise, non-parametric tests were used: Mann–Whitney U test and the Kruskal–Wallis test (for more than two groups). We performed paired tests to evaluate the ROTEM components for the patients at various time points (paired *t*-test or Wilkoxon signed-ranked test). The correlation between the WHO clinical progression scale and ROTEM parameters was investigated by calculating Spearman’s co-efficient.

“Since an in-hospital mortality of approximately 10% was observed at our institution during the study period [15] a power analysis was implemented in order to determine the population required for a statistically significant difference of 5% between patients exhibiting hypercoagulable and normal/hemorrhagic ROTEM, respectively. Power analysis was performed using G*Power software (version 3.1, Düsseldorf, Germany) [16] by setting the following parameters: α = 0.05, statistical power = 0.8, effect size index for the chi-square test (w) = 0.3”. It turns out that a total sample size of 143 patients is required to demonstrate significance at a 5% difference in mortality between the two groups.

In multivariable analysis co-variates that have been associated with a poor prognosis and the development of COVID-19 sequelae, such as age, gender, the need for supplemental oxygen anytime throughout hospital stay, the Charlson Comorbidity Index (CCI) and body mass index (BMI) were used [17,18,19,20]. Multivariable analysis was performed using the logistic regression approach to investigate the following: (1) whether the presence of a previously hypercoagulable ROTEM was associated with the persistence of symptoms at one month after discharge. For this, we used two models, both having the presence of these symptoms at one month post-discharge, as dependent variables. In the first model, hypercoagulable ROTEM at enrollment, and in the second one, hypercoagulable ROTEM at the time of discharge, were used as independent variables with age, gender, the need for supplemental oxygen anytime throughout hospital stay, CCI and BMI serving as co-variates; (2) whether the presence of a previously hypercoagulable ROTEM was related to the persistence of symptoms three months after discharge. We used three models having the presence of these symptoms at three months post-discharge as dependent variables. Hypercoagulable ROTEM at the time of enrollment, at the time of discharge and at one month post discharge were used as independent variables in the three different models, with age, gender, the need for supplemental oxygen anytime throughout hospital stay, CCI and BMI serving as co-variates; (3) whether a previously hypercoagulable ROTEM was linked to the presence of an abnormal DLco at three months after discharge. For this purpose, we used two models having the DLco at three months as dependent variables and age, gender, the need for supplemental oxygen anytime throughout hospital stay, smoking history and hypercoagulable ROTEM at enrollment and at the time of discharge were used as independent variables. In general, aiming to avoid multicollinearity in the multiple logistic regression model (due to the significant interdependence of the individual ROTEM indices), we chose to treat only the spherical ROTEM profile as an independent variable, even when other ROTEM indices were significantly associated with clinical features in the univariate analysis. A G-squared Log-likelihood test was used to evaluate the goodness-of-fit. Missing data were handled using a listwise case deletion approach. Thus, 44 participants were evaluated by the logistic regression models (the number of patients who completed clinical evaluation and pulmonary function testing and DLco measurements after hospital discharge). For every logistic regression analysis, the degrees of freedom for the intercept model (*n* − 1) and for the selected model (*n* − *k* − 1) are depicted alongside the area under the receiver–operator characteristic curve (AU-ROC). *N* represents the number of participants and *k* represents the number of the selected variables. The significance level (*p*-value) for the study was set to 0.05 and all tests were two-sided.

### 2.4. Patient and Public Involvement Statement

The study was designed and largely conducted in the middle of the pandemic, in a condition characterized by a restriction in social life, including strict lockdowns. It was therefore not possible to involve the patients or the public in the design, or conducting, or reporting, or dissemination plans of our research.

## 3. Results

### 3.1. Study Population

The study was prematurely terminated because our team was assigned to take care of non-COVID-19 patients as a rotational system for hospital personnel between COVID-19 and non-COVID-19 services was adopted sometime during the pandemic. Therefore, our final study population consisted of 62 patients hospitalized due to COVID-19-related pneumonia, with a mean age (±standard error of the mean) of 59.3 (±1.8), and a female/male ratio of 1.6. A total of 9/62 patients had hyper-cholesterolaemia and/or hyper-triglyceridaemia, but they were under dietary and pharmacological treatment and in general, their dislipidaemia was well controlled. None of the patients reported a previous COVID-19 infection. A total of 13/62 patients had received at least one dose of a SARS-CoV-2 vaccine (first generation mRNA or adeno-viral vector) prior to the onset of their illness, but only 2 had completed their vaccination scheme, which protects from severe COVID-19 outcomes and, potentially, from long COVID-related abnormalities [21]. The rest of the epidemiological/clinical and laboratory characteristics are depicted in Supplementary Table S2A and Table S2B, respectively. Patients received pharmacological treatment and respiratory support according to the national guidelines for COVID-19. The patients were treated according to the Greek National Public Health Organization guidelines for COVID-19 [eody.gov.gr/en/covid-19/ (accessed on 27 August 2020)]. Sixty of them received a prophylactic dose of low-molecular-weight-heparin (LMWH) or fondaparinux, one patient was under 5 mg of apixaban, twice daily, due to atrial fibrillation, and one patient did not receive any anticoagulant due to hematuria. LMWH or fondaparinux were given up to the discharge day. Apart from the patient who presented myocardial infraction post-discharge (see below) and received aspirin, no other patient received drugs that could be suspected to affect ROTEM findings, including nonsteroidal anti-inflammatory drugs (NSAIDs), E-EPA, daily use of fish oil, or antihemorrhagic drugs, during their participation in the study. No clinically evident thrombotic event was observed in any patient during their hospital stay.

The first (enrollment) sample for ROTEM was obtained at the median (IQR) 2 (1–3) days after admission and 9 (7–11) days after symptom onset. A total of 12 patients presented clinical progression, 7/12 deceased and 5/12 recovered. Fifty-five patients were eventually discharged from the hospital. No thromboembolic or bleeding events (due to anticoagulant treatment) were observed during hospitalization. Two weeks after discharge, two patients were readmitted to the hospital, the first because of catheter-related bloodstream infection and septic shock (the patient died), and the other one with myocardial infarction (this patient was discharged again). Finally, 44 patients were present at the follow-up visits, one and three months post-discharge, including all 5 patients who experienced progression during their hospital stay (Supplementary Figure S1).

### 3.2. Association Between ROTEM Profile and Clinical Severity and Progression During Hospitalization

At enrollment, ROTEM was normal in 32 patients (51.6%), hypercoagulable in 29 (46.7%) patients and hemorrhagic in 1 patient (1.6%). Among the 12 patients who presented clinical deterioration, 6 had a hypercoagulable ROTEM at enrollment, which remained hypercoagulable, and 4 of them at deterioration (for the other 2 cases, there were no available data because the patients progressed rapidly and died before sampling was possible). All five patients with an initially normal ROTEM and subsequent clinical progression presented a hypercoagulable profile at the time of clinical deterioration. The hemorrhagic case remained at the same status at deterioration. A hypercoagulable ROTEM was more common at the time of clinical deterioration (9/10 patients) than at enrollment (29/62 patients, *p* = 0.02). A hypercoagulable ROTEM at enrollment was not significantly associated with subsequent clinical deterioration, which occurred in 6/29 patients with a hypercoagulable enrollment status and in 6/33 patients with a non-hypercoagulable enrollment status (*p* = 0.08). Upon discharge, ROTEM was conducted in 55 patients (7 patients had died). Among the 32 patients who had normal enrollment ROTEM, the discharge ROTEM was hypercoagulable in 4 and normal in 26. Two of the patients with an initially normal ROTEM died and discharge ROTEM data were not available. At discharge, among the 29 patients with hypercoagulable enrollment ROTEM, it remained hypercoagulable in 15 (51.7%) and was normalized in 9 (31%) (5 of them had died) (*p* = 0.016; Odds Ratio: 0.22, 95% CI: 0.06–0.75, for a hypercoagulable ROTEM profile to remain hypercoagulable upon discharge).

We then investigated possible links between individual ROTEM parameters and the clinical severity (determined by the 11-point WHO CPS) at the time of enrollment, during deterioration and at discharge (Supplementary Table S3). At enrollment, CFT values were negatively correlated while Alp and A10 values were positively correlated with the CPS score. At progression, Alp and A10 values were positively correlated with the CPS score. At discharge, A10 and A20 values were positively correlated with the CPS score (Supplementary Table S3). The values of ROTEM parameters linked with fibrinolysis (Li30, Li60) were not correlated with the WHO severity score. Overall, the above observations demonstrate a link between the clinical severity and the velocity of clot formation and its stability, but not with fibrinolysis.

### 3.3. Association Between ROTEM Profile and Survival

We then attempted to define whether performing ROTEM at enrollment could predict the hospital mortality. Hospital death occurred in 5/29 patients with a hypercoagulable profile and in 2/33 patients with a non-hypercoagulable profile at enrollment (*p* = 0.2). However, the study was not powered to uncover significant differences in mortality between the two groups since for this purpose, we needed to enroll 143 patients. Patients who died had higher enrollment levels of A10, A20 and Alp compared to those who survived (Supplementary Table S4), implying a link to accelerated clot formation. Thrombocytopenia and increased MPV at enrollment were also related to death (Supplementary Table S4).

### 3.4. Hypercoagulable ROTEM Profile Was Significantly Associated with Long COVID Abnormalities

#### 3.4.1. Association Between ROTEM Profile and Clinical Symptoms After Discharge

At the follow-up visits, 44 out the 54 discharged patients were present. The patients who were present at the follow-up did not differ from those who were not present in terms of age, gender, smoking history, obesity, Charlson Comorbidity Index, the need for supplemental oxygen or clinical progression during hospital stay. One month after discharge, 16 (36.3%) of the patients had at least one symptom (fatigue, dyspnea, hair loss, anxiety, ageusia, memory disorders, palpitations), 9/44 patients experienced exertional dyspnea and 9/44 experienced fatigue. One patient, who had a normal ROTEM at enrollment and at discharge, was re-admitted with myocardial infarction and the ROTEM was hypercoagulable at the time of the second admission. At three months, 9/44 had exertional dyspnea, 2/44 had fatigue and in total, 10/44 (22.7%) had at least one symptom. Thus, we asked whether a hypercoagulable ROTEM profile during hospitalization or the early post-discharge period is associated with post-acute COVID-19 symptoms at later time points.

Symptomatic patients at one month after discharge were more likely to present a hypercoagulable ROTEM at enrollment, at discharge and at one month after discharge, than those with no symptoms at this time point (Supplementary Table S5). Similarly, symptomatic patients at three months were more likely to present a hypercoagulable ROTEM at enrollment and one month after discharge (Supplementary Table S6). When we searched for links between persistent symptoms and previously observed abnormalities of specific ROTEM parameters, we found that patients with symptoms at one month post-discharge were characterized by higher A10, A20, Alp, MCF values at discharge compared to those without symptoms at this time point (Supplementary Table S7). Similarly, at three months post-discharge, symptomatic patients were characterized by higher one-month levels of A10, A20, Alp, MCF and lower levels of CFT compared to the asymptomatic ones (Supplementary Table S8), suggesting an association between post-acute COVID-19 symptoms and rapid clotting, enhanced clot firmness or increased clot propagation that was present several weeks prior to symptom evaluation.

In the multivariate analysis, the persistence of symptoms at 1 month post-discharge was significantly related to a hypercoagulable ROTEM profile at discharge and the female gender (Table 1).

When we evaluated symptoms at 3 months post-discharge, we observed that the BMI variable was characterized by a perfect separation and thus, it was removed from the final models. Due to a low goodness-of-fit (non-significant *p*-value for G-squared Log-likelihood ratio test), we rejected the first two models (as they were described in the Methods section) and developed a single model where symptoms at 3 months were treated as the dependent variable, ROTEM at 1 month was considered as the independent variable and age, gender, the need for supplemental oxygen during hospitalization and CCI were treated as covariates. Thus, we observed that a hypercoagulable ROTEM profile at 1 month was independently associated with the presence of symptoms at 3 months after discharge (Table 2).

#### 3.4.2. Relation of ROTEM Parameters with DLco at Three Months

Patients with hypercoagulable ROTEM at enrollment, at discharge and at one month after discharge, were more likely to have an abnormal DLco (<80% of the predicted value) at three months after discharge (Table 3). Patients with an abnormal DLco at three months after discharge, were characterized by higher A20, MCF and platelet enrollment values, higher A10, A20, Alp, MCF, platelet and decreased CFT discharge values, as well as higher A20, Alp, MCF and lower CFT values at 1 month after discharge, compared to the patients with normal DLco at three months after discharge (Supplementary Table S9), which is suggestive of a link between abnormal DLco and increased clot firmness, accelerated clotting and increased strength of the thrombus, all observed several weeks before DLco evaluation. The multivariate analysis demonstrated that hypercoagulable ROTEM profile at enrollment and at discharge and the female gender could predict the presence of abnormal DLco three months after discharge (Table 4). We did not find any link between forced vital capacity (FVC % of the predicted value), lung diffusion capacity/alveolar ventilation (DLco/VA % of the predicted value) and alveolar ventilation (VA, % of predicted) determined three months after discharge and the previous or synchronous ROTEM profile (Supplementary Tables S10–S12).

## 4. Discussion

We investigated herein the potential links between hypercoagulable ROTEM, clinical severity and the outcome of patients with COVID-19-related pneumonia during their hospitalization and post-discharge follow-up, focusing on the potential ability of ROTEM to predict the course of the disease. Our main findings were the following: (a) a hypercoagulable ROTEM profile was more common at the time of clinical deterioration than at enrollment and the levels of certain hypercoagulable ROTEM indices correlated with the values of the clinical severity score; (b) hypercoagulable ROTEM at enrollment was not associated with subsequent clinical progression or in-hospital death; (c) patients with hypercoagulable ROTEM at discharge and one month after discharge presented an increased risk of presenting persistent symptoms 1 and 3 months after discharge; (d) patients with hypercoagulable ROTEM at enrollment, at discharge, and 1 month after discharge, were more likely to have an abnormal lung diffusion capacity 3 months after discharge; (e) high levels of certain hypercoagulable ROTEM indices were associated with an increased risk of symptom persistence at later stages of the disease; and (f) the multivariate analysis demonstrated strong associations between (i) the female gender or hypercoagulable ROTEM at discharge and the presence of symptoms at 1 month post-discharge, (ii) hypercoagulable ROTEM at 1 month after discharge and the presence of symptoms at 3 months post-discharge, and (iii) hypercoagulable ROTEM at enrollment and at discharge or female gender and an impaired DLco value (<80% of predicted) at 3 months after discharge.

This is the first study investigating the clinical utility of ROTEM in non-ICU patients, who were hospitalized due to COVID-19, a disease characterized by a hypercoagulable state [22,23,24]. We observed a link between hypercoagulable ROTEM indices and concomitant disease severity and an increased probability of hypercoagulable state occurrence at the time of clinical deterioration than during the earlier baseline evaluation. These findings are in line with the previously described association between endothelial dysfunction and procoagulant state with COVID-19 severity [25,26,27]. On the contrary, a baseline hypercoagulable ROTEM profile did not predict either disease progression or hospital death, adding to the debate on whether or not ROTEM predicts the clinical outcome of COVID-19 disease during hospitalization [25,28,29,30]. The reasons for conflicting results of different studies may reflect variations in patients’ characteristics or differences at the disease stage at the time of blood sampling [31]. However, it is possible, that the absence of significant relations between global hypercoagulable ROTEM profiles and death observed in the present study might be attributed to the small number of observations. In fact, when we focused on the link between hospital death and the levels of certain hypercoagulable ROTEM indices, we found that the patients who died exhibited increased baseline A10, A20, Alp, MCF and decreased CT (showing overall an accelerated clot formation and high clot strength) values. Whether a baseline ROTEM possesses predictive utility for in-hospital outcomes of patients with COVID-19-related pneumonia requires further investigation.

We, in this study, for the first time, observed a link between hypercoagulable ROTEM profiles at different time points during hospitalization or during the early post-hospitalization period and long-lasting symptoms, which present a major problem affecting millions of COVID-19 survivors [3,5,6,32]. Multiple logistic regression models demonstrated strong associations between hypercoagulable ROTEM at discharge and the presence of symptoms at one month after discharge, as well as hypercoagulable ROTEM at one month after discharge and the presence of symptoms at three months after discharge. A significant proportion of COVID-19 survivors, who were hospitalized for severe acute disease, exhibit an impaired oxygen lung diffusion capacity, an abnormality that may last up to 2 years after the initial SARS-CoV-2 infection [33]. Previous reports indicated that severely impaired DLco is not necessarily linked to fibrotic lesions in high-resolution chest computed tomography, suggesting that different mechanisms may underlie this abnormal finding [34]. In this regard, we demonstrated, for the first time, that patients with impaired DLco three months after discharge were more likely to have a hypercoagulable ROTEM global profile at the time of enrollment or at discharge, along with an increased mean platelet volume (suggesting the presence of, for the most part, “large” platelets) at 1 month post-discharge. The multivariate analysis showed that only a hypercoagulable ROTEM profile at enrollment and at discharge was independently linked to low DLco at three months post-discharge. The association between impaired lung diffusion capacity and hypercoagulation or increased platelet size (both connected with the risk of micro-vascular obstruction) supports the notion that a dysfunctional or damaged pulmonary capillary bed is a major factor of impaired lung function, observed at the context of post-acute-COVID-19 [3,4,35,36]. From a clinical viewpoint, the probable predictive role of ROTEM for persistent symptoms and decreased DLco after the acute phase of COVID-19 pneumonia is of major importance and requires validation. Being able to predict who, among hospitalized patients with COVID-19-related pneumonia, will suffer with post-acute-COVID-19 sequelae would permit the optimal prioritization of patients and individualization of follow-up programs, which would increase the efficacy of long-COVID-19 services. However, the findings of the present study are merely exploratory and a potential predictive role of ROTEM requires further investigation.

Several limitations of the present study deserve a comment: (1) this is a single-center study that was prematurely terminated and therefore, the sample size is small and we were not able to reach a conclusion for the link between ROTEM findings and hospital mortality; (2) the lack of a non-COVID-19 control group, such as patients with community-acquired pneumonia, who may also present a long symptomatic post-discharge period; (3) the fact that we were not able to perform ROTEM on weekends or on public holidays, so enrollment or deterioration samples could be only obtained on the first working day after the patient’s initial consent or clinical deterioration, a condition that led to the loss of the “deterioration” sample from two patients who rapidly progressed to death; (4) a significant proportion of patients who survived the acute disease did not appear at the follow-up visits after discharge. On the other hand, a noticeable methodological advantage (apart from its prospective nature) of the study is that we analyzed blood samples obtained at pre-defined, clinically relevant time points (i.e., deterioration, discharge, follow-up visits) instead of using random samples obtained sometimes during the hospital stay of the patients. This design gave us the opportunity to capture the dynamic nature of the COVID-19 disease and explore the possible predictive potential of ROTEM for post-hospitalization outcomes.

In conclusion, we demonstrated here that the clinical progression of COVID-19 pneumonia occurs in parallel with the further deterioration of hemostasis towards a hypercoagulable state, as well as that a hypercoagulable ROTEM profile may predict the presence of symptoms and respiratory functional impairment that persist for weeks after discharge. Moreover, from a pathobiological viewpoint, our findings support the notion that hypercoagulability plays an important role, not only during the acute phase of COVID-19 pneumonia, but also in clinical disorders persisting after discharge. Due to the methodological limitations discussed above, our study should be considered exploratory rather than conclusive. However, our findings pave the way for future studies that may attempt to clarify the role of persistent microvascular thrombosis in the long-COVID pathogenesis and to validate our observations on the predictive role of ROTEM profiles for post-acute-COVID abnormalities.

## Figures and Tables

**Table 1:** Multivariable analysis exploring the link between the presence of symptoms one month post-discharge and ROTEM profiles at previous time points. Model A: degrees of freedom for the intercept-only model = 43 and for the selected model = 37, AU-ROC (CI) = 0.84 (0.73–0.97), *p* < 0.001. Model B: degrees of freedom for the intercept-only model = 43 and for the selected model = 37, AU-ROC (CI) = 0.90 (0.8–0.99), *p* < 0.001.

Independent Variables	Odds Ratio	95% CI	*p*
Model A, ROTEM at enrollment			
Intercept	0.04	0.001–5.3	0.24
Age (per year)	1.03	0.93–1.14	0.62
Gender (Female vs. Male)	7.66	1.54–49.81	0.02
Need for O_2_ during hospital stay (No vs. Yes)	1.47	0.12–3.69	0.66
ROTEM at enrollment (Hypercoagulable vs. Νon-hypercoagulable)	4.4	0.84–27.17	0.09
BMI > 30 Kg/m^2^ (Yes vs. No)	0.53	0.02–4.94	0.61
CCI (>2 vs. ≤2)	0.48	0.03–5.83	0.58
Model B, ROTEM at discharge			
Intercept	0.02	0.001–5.24	0.2
Age (per year)	1.01	0.9–1.13	0.92
Gender (Female vs. Male)	26.05	3.31–649.2	<0.01
Need for O_2_ during hospital stay (No vs. Yes)	1.41	0.24–8.83	0.7
ROTEM at discharge (Hypercoagulable vs. Νon-hypercoagulable)	22.37	3.16–534.4	<0.01
BMI > 30 Kg/m^2^ (Yes vs. No)	0.26	0.03–7.52	0.49
CCI (>2 vs. ≤2)	0.68	0.03–11.55	0.79

CI—confidence interval; CCI—Charlson Comorbidity Index; BMI—body mass index; AU-ROC—area under the receiver–operator characteristic curve.

**Table 2:** Multivariable analysis exploring the link between the presence of symptoms three months after discharge and ROTEM one month post-discharge. Degrees of freedom for the intercept-only model = 43 and for the selected model = 38, AU-ROC (CI) = 0.86 (0.74–0.98), *p* < 0.001.

Independent Variables	Odds Ratio	95% CI	*p*
ROTEM one month post-discharge			
Intercept	1.38	0.009–250.3	0.9
Age (per year)	0.93	0.83–1.03	0.25
Gender (Male vs. Female)	6.23	0.86–66.49	0.09
Need for O_2_ during hospital stay (No vs. Yes)	2.1	0.3–16.53	0.45
ROTEM one month post-discharge (Hypercoagulable vs. Νon-hypercoagulable)	32.28	3.81–515.8	<0.01
CCI (>2 vs. ≤2)	1.08	0.06–21.05	0.06

CI—confidence interval; CCI—Charlson Comorbidity Index; AU-ROC—area under the receiver–operator characteristic curve.

**Table 3:** Link between lung diffusion capacity (DLco) determined three months after discharge and the previous or synchronous ROTEM profile (non-thrombotic = normal/hemorrhagic).

	DLco				
	Normal	Decreased	Total	*p*	OR (95% CI)
ROTEM At Enrollment				<0.001	17.5 (3.4–84.5)
Non-hypercoagulable	20	8	28		
Hypercoagulable	2	14	16		
Total	22	22	44		
ROTEM At Discharge					
Non-hypercoagulable	20	8	28	<0.001	17.5 (3.4–84.5)
Hypercoagulable	2	14	16		
Total	22	22	44		
ROTEM one month after discharge					
Non-hypercoagulable	22	15	37	0.04	21.8 (1.17–410)
Hypercoagulable	0	7	7		
Total	22	22	44		

Normal DLco values are those ≥80% of the predicted value. CI—confidence interval.

**Table 4:** Multivariable analysis exploring the link between lung diffusion capacity (DLco) determined three months after discharge and ROTEM profiles at enrollment and at discharge. Normal DLco values are those ≥ 80% of the predicted value. Model A: degrees of freedom for the intercept-only model = 43 and for the selected model = 37, AU-ROC (CI) = 0.86 (0.75–0.97), *p* < 0.001. Model B: degrees of freedom for the intercept-only model = 43 and for the selected model = 37, AU-ROC (CI) = 0.87 (0.76–0.98), *p* < 0.001.

Independent Variables	Odds Ratio	95% CI	*p*
Model A, ROTEM at enrollment			
Intercept	0.43	0.003–40.42	0.72
Age (per year)	0.99	0.91–1.09	0.98
Gender (Female vs. Male)	3.00	0.46–24.43	0.26
Need for O_2_ during hospital stay (No vs. Yes)	0.62	0.12–3.06	0.56
ROTEM at enrollment (Hypercoagulable vs. Non-hypercoagulable)	17.00	2.95–172.7	<0.01
CCI (>2 vs. ≤2)	0.64	0.06–6.13	0.7
Smoking (Yes vs. No)	0.78	0.08–6.11	0.81
Model B, ROTEM at discharge			
Intercept	1.59	0.01–157.1	0.84
Age (per year)	0.96	0.87– 1.05	0.42
Gender (Female vs. Male)	9.02	1.32–92.60	0.04
Need for O_2_ during hospital stay (No vs. Yes)	0.39	0.06–2.03	0.28
ROTEM at discharge (Hypercoagulable vs. Non-hypercoagulable)	27.96	4.61–316.6	<0.01
CCI (>2 vs. ≤2)	1.33	0.12–16.27	0.81
Smoking (Yes vs. No)	1.07	0.10–10.11	0.95

CI—confidence interval; CCI—Charlson Comorbidity Index; AU-ROC—area under the receiver–operator characteristic curve.

## Data Availability

Data are available upon contact with the corresponding author.

## Supplementary Materials

The following supporting information can be downloaded at: https://www.mdpi.com/article/10.3390/v16121916/s1, Figure S1: Clinical trajectories of the study COVID-19 patients title; Table S1: Normal ranges of ROTEM parameters. CT, clotting time; CFT, Clot formation time; MCF, Maximum clot firmness; A10 and A20, Amplitudes at 10 and 20 min respectively; Alp, α-angle; LI30 and LI60, the percentage of thrombus lysis in 30 and 60 min respectively. ROTEM was characterized as hypercoagulable when at least one of the following were present: CFT and/or CT were below the normal range, alpha angle (α), maximum clot firmness (MCF), A10, A20 were above the normal range. ROTEM was characterized as hemorrhagic if CT and/or CFT were above the normal range; Table S2A: Patients’ epidemiological/clinical characteristics. Quantitative variables are depicted as mean ± standard error of the mean (SEM). Qualitative variables are depicted as numbers and percentages. BMI: Body Mass Index. ROTEM: Rotational Thromboelastometry; Table S2B: Patients’ laboratory characteristics (enrollment). Quantitative variables are depicted as mean ± standard error of the mean (SEM) if they were normally distributed or as median (inter-quartile range—IQR) if they were not normally distributed. Hb, hemoglobin; WBCs, white blood cells; MPV, mean platelet volume; aPTT, activated partial thromboplastin time; INR, international normalized ratio; CT, clotting time; CFT, Clot formation time; MCF, Maximum clot firmness; A10 and A20, Amplitudes at 10 and 20 min respectively; Alp, α-angle; LI30 and LI60, the percentage of thrombus lysis in 30 and 60 min respectively; Table S3: Correlation between WHO clinical progression scale and ROTEM parameter values at different time points during hospital stay. CT, clotting time; CFT, Clot formation time; MCF, Maximum clot firmness; A10 and A20, Amplitudes at 10 and 20 min respectively; Alp, α-angle; LI30 and LI60, the percentage of thrombus lysis in 30 and 60 min respectively; Alp, α-angle; LI30 and LI60, the percentage of thrombus lysis in 30 and 60 min respectively. R = Spearman’s co-efficient; Table S4: Link between hospital mortality and coagulation test or ROTEM parameter values at enrolment. Quantitative variables are depicted as mean ± standard error of the mean (SEM) and they were analysed by Student’s *t*-test, if they were normally distributed. Quantitative variables are depicted as median (inter-quartile range—IQR) and they were analysed by Mann-Whitney U-test if they were not normally distributed. *p*-values < 0.05 were considered significant. aPTT, activated partial thromboplastin time; INR, international normalized ratio; MPV, mean platelet volume; CT, clotting time; CFT, Clot formation time; MCF, Maximum clot firmness; A10 and A20, Amplitudes at 10 and 20 min respectively; Alp, α-angle; LI30 and LI60, the percentage of thrombus lysis in 30 and 60 min respectively; Table S5: Link between symptoms at one month after discharge and the previous or synchronous ROTEM profile (non-hypercoagulable = normal/hemorrhagic); Table S6: Link between symptoms at three months after discharge and previous or synchronous ROTEM profile (non-hypercoagulable = normal/hemorrhagic); Table S7: Link between the presence of symptoms at one month after discharge and previous coagulation test or ROTEM parameter values. Quantitative variables are depicted as mean ± standard error of the mean (SEM) and they were analysed by Student’s *t*-test, if they were normally distributed. Quantitative variables are depicted as median (inter-quartile range—IQR) and they were analysed by Mann-Whitney U-test if they were not normally distributed. *p*-values < 0.05 were considered significant. aPTT, activated partial thromboplastin time; INR, international normalised ratio; MPV, mean platelet volume; CT, clotting time; CFT, Clot formation time, MCF, Maximum clot firmness; A10 and A20, Amplitudes at 10 and 20 min respectively; Alp, α-angle; LI30 and LI60, the percentage of thrombus lysis in 30 and 60 min respectively; Table S8: Link between the presence of symptoms at three months after discharge and previous coagulation test or ROTEM parameter values. Quantitative variables are depicted as mean ± standard error of the mean (SEM) and they were analysed by Student’s *t*-test, if they were normally distributed. Quantitative variables are depicted as median (inter-quartile range—IQR) and they were analysed by Mann-Whitney U-test if they were not normally distributed. *p*-values < 0.05 were considered significant. aPTT, activated partial thromboplastin time; INR, international normalised ratio; MPV, mean platelet volume; CT, clotting time; CFT, Clot formation time; MCF, Maximum clot firmness; A10 and A20, Amplitudes at 10 and 20 min respectively; Alp, α-angle; LI30 and LI60, the percentage of thrombus lysis in 30 and 60 min respectively; Table S9: Link between the lung diffusion capacity (DLco) determined at three months after discharge and previous coagulation test or ROTEM parameter values. Quantitative variables are depicted as mean ± standard error of the mean (SEM) and they were analysed by Student’s *t*-test, if they were normally distributed. Quantitative variables are depicted as median (inter-quartile range—IQR) and they were analysed by Mann-Whitney U-test if they were not normally distributed. Normal DLco values are those >80% of the predicted value. aPTT, activated partial thromboplastin time; INR, international normalised ratio; MPV, mean platelet volume; CT, clotting time; CFT, Clot formation time, MCF, Maximum clot firmness; A10 and A20, Amplitudes at 10 and 20 min respectively; Alp, α-angle; LI30 and LI60, the percentage of thrombus lysis in 30 and 60 min respectively; Table S10: Link between forced vital capacity (FVC % of the predicted value) determined three months after discharge and the previous or synchronous ROTEM profile (non-hypercoagulable = normal/hemorrhagic). FVC > 80% of the predicted value is considered normal. Comparisons were made using Fisher’s exact test; Table S11: Link between lung diffusion capacity/alveolar ventilation (DLco/VA % of the predicted value) determined three months after discharge and the previous or synchronous ROTEM profile (non-hypercoagulable = normal/hemorrhagic). DLco/VA > 80% of the predicted value is considered normal. Comparisons were made using Fisher’s exact test; Table S12: Link between alveolar ventilation (VA, % of predicted value) determined three months after discharge and the previous or synchronous ROTEM profile (non-hypercoagulable = normal/hemorrhagic). Comparisons were made using Fisher’s exact test.

## Abbreviations

COVID-19: Coronavirus disease 2019, DLco: Diffusing capacity for carbon monoxide, ROTEM: Rotational thromboelastometry, SARS-CoV-2: Severe acute respiratory syndrome coronavirus 2, CCI: Charlson’s comorbidity index, BMI: Body mass index.
