# Supplementary material for: Hypercoagulable Rotational Thromboelastometry During Hospital Stay Is Associated with Post-Discharge DLco Impairment in Patients with COVID-19-Related Pneumonia
Source: Viruses. 2024 Dec 14;16(12):1916. doi: 10.3390/v16121916 (PMC11680182; doi:10.3390/v16121916)
Supplement: Supplementary file 1 [file viruses-16-01916-s001.zip › viruses-3326281-supplementary.pdf]

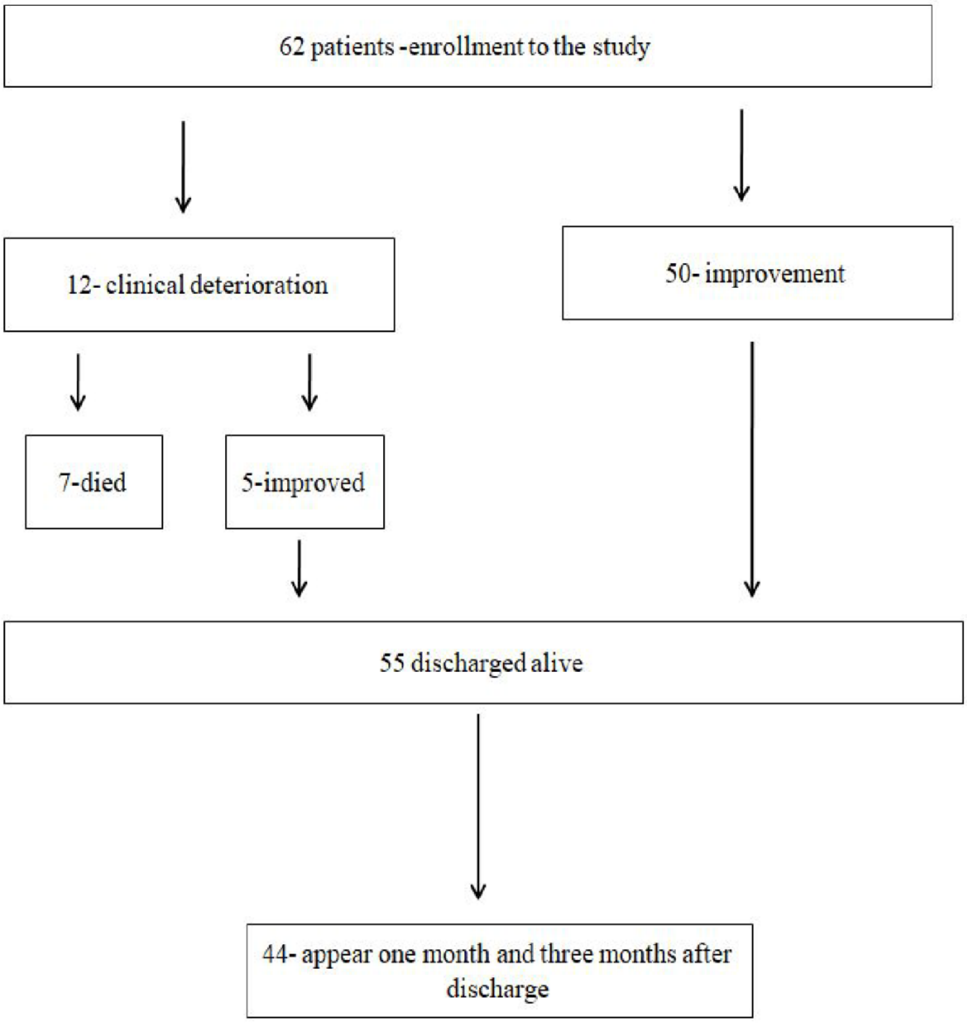

Figure S1: Clinical trajectories of the study COVID-19 patients

Supplementary Table S1: Normal ranges of ROTEM parameters. CT, clotting time; CFT, Clot formation time; MCF, Maximum clot firmness; A10 and A20, Amplitudes at 10 and 20 minutes respectively; Alp, α-angle; LI30 and LI60, the percentage of thrombus lysis in 30 and 60 minutes respectively**.** ROTEM was characterized as hypercoagulable when at least one of the following were present: CFT and/or CT were below the normal range, alpha angle (α), maximum clot firmness (MCF), A10, A20 were above the normal range. ROTEM was characterized as hemorrhagic if CT and/or CFT were above the normal range.

ROTEM parameters	Normal range	Hypercoagulable	Hemorrhagic
CT, sec	300-999	300	>999
CFT, sec	150-700	<150	>700
Alp, degrees	30-70	>70	
A10, mm	25-55	>55	
A20, mm	35-60	>20	
MCF, sec	40-65	>65	
Li30, %	>15		
Li60, %	>15		

Supplementary Table S2A: Patients' epidemiological/clinical characteristics. Quantitative variables are depicted as mean ± standard error of the mean (SEM). Qualitative variables are depicted as numbers and percentages. BMI: Body Mass Index. ROTEM: Rotational Thromboelastometry.

Patients' epidemiological/clinical characteristics	n=62
Age in years	59.3 ±1.8
Female/Male ratio	1.6
Obesity (BMI ≥ 30 kg/m^2)	10 (16%)
Need for Supplemental Oxygen	42 (68%)
Charlson Comorbidity Index (>2)	27 (43.5%)
Smoking/ex-smokers	19 (31%)
Clinical progression	12 (19.4%)
Intubation during hospitalization	5 (8%)
In-hospital mortality	7 (11.3%)
ROTEM at enrolment	
Normal	32 (51.6%)
Thrombotic	29 (46.8%)
Hemorrhagic	1 (1.6%)

Supplementary Table S2B: Patients' laboratory characteristics (enrollment). Quantitative variables are depicted as mean ± standard error of the mean (SEM) if they were normally distributed or as median (inter-quartile range - IQR) if they were not normally distributed. Hb, hemoglobin; WBCs, white blood cells; MPV, mean platelet volume; aPTT, activated partial thromboplastin time; INR, international normalized ratio; CT, clotting time; CFT, Clot formation time; MCF, Maximum clot firmness; A10 and A20, Amplitudes at 10 and 20 minutes respectively; Alp, α-angle; LI30 and LI60, the percentage of thrombus lysis in 30 and 60 minutes respectively.

Patients' Laboratory characteristics	
Hb, gr/dL	13.2 ±0.22
WBCs, x 10^3 /μL	7.1 (5.5-10.1)
Lymphocytes, x 10^3/μL	1.06 (0.73-1.56)
Platelets, x 10^3/μL	199 (164-288)
MPV, fL	10.7 (10.1-11.3)
D-Dimers, μg/mL	0.78 (0.48 - 1.43)
Fibrinogen, mg/dL	597 ±20.4
aPTT, sec	32.2 (29.2 - 36.4)
INR	1.06 (1-1.15)
Ferritin, ng/ml	423.5 (202-639)
Troponin, pg/ml	8 (4.5 - 15)
CRP, mg/dL	6 (3.8-10.2)
ROTEM parameters	
CT, sec	532 (454-686)
CFT, sec	192 (133-281)
Alp, degrees	54.3 ±1.55
A10, mm	45.6 ±1.45
A20, mm	56.4 ±1.2
MCF, sec	59.4 ±1.08
Li30, %	100 (100-100)
Li60, %	93 ± 0.55

Supplementary Table S3: Correlation between WHO clinical progression scale and ROTEM parameter values at different time points during hospital stay. CT, clotting time; CFT, Clot formation time; MCF, Maximum clot firmness; A10 and A20, Amplitudes at 10 and 20 minutes respectively; Alp, α-angle; LI30 and LI60, the percentage of thrombus lysis in 30 and 60 minutes respectively. R= Spearman's co-efficient.

ROTEM parameters	Enrollment		Progression		Discharge	
	R	p	r	p	r	p
CT, sec	-0.20	0.10	-0.44	0.19	0.09	0.49
CFT, sec	-0.25	**0.04**	-0.62	0.05	-0.10	0.44
Alp, degrees	0.27	**0.03**	0.74	**0.01**	0.19	0.16
A10, mm	0.27	**0.02**	0.55	0.09	0.27	**0.04**
A20, mm	0.22	0.07	0.42	0.21	0.30	**0.02**
MCF, sec	0.14	0.24	0.13	0.70	0.23	0.07
Li30, %	0.02	0.82	-0.36	0.29	0.08	0.51
Li60, %	0.03	0.80	-0.54	0.10	0.08	0.54

Supplementary Table S4: Link between hospital mortality and coagulation test or ROTEM parameter values at enrolment. Quantitative variables are depicted as mean ± standard error of the mean (SEM) and they were analysed by Student's t-test, if they were normally distributed. Quantitative variables are depicted as median (inter-quartile range - IQR) and they were analysed by Mann-Whitney U-test if they were not normally distributed. P-values <0.05 were considered significant. aPTT, activated partial thromboplastin time; INR, international normalized ratio; MPV, mean platelet volume; CT, clotting time; CFT, Clot formation time; MCF, Maximum clot firmness; A10 and A20, Amplitudes at 10 and 20 minutes respectively; Alp, α-angle; LI30 and LI60, the percentage of thrombus lysis in 30 and 60 minutes respectively.

	Dead (n=7)	Alive (N=55)	p
Platelets, $x10^3$ /μL	150 (130-183)	216 (167-290)	**0.02**
aPTT, sec	34.8 (33-37.7)	31.4 (28.7-36.3)	0.06
D_Dimers, μg/mL	0.85 (0.63-1.65)	0.76 (0.46-1.42)	0.13
INR	1 ±1-1.2	1.1 ±1-1.2	0.89
MPV, fL	11.7 (10.8-11.8)	10.5 (10-11.2)	**<0.01**
A10, mm	53.7 ±3.9	44.5 ±1.51	**0.04**
A20, mm	63.3 ±8.06	55.6 ±1.25	**0.04**

Alp, degrees	63 ±4.99	53.2 ±1.58	**0.04**
CFT, sec	146 (76-308)	195 (144-277)	0.16
CT, sec	410 (171-701)	535 (457-685)	0.09
Li30, %	100 (100-100)	100 (100-100)	0.63
Li60, %	94.7 ±1.27	92.6 ±0.6	0.24
MCF, sec	65.3 ±2.9	58.6 ±1.13	0.05

Supplementary Table S5: Link between symptoms at one month after discharge and the previous or synchronous ROTEM profile (non- hypercoagulable =normal/hemorrhagic).

	**Symptoms at one month after discharge**				
	**No**	**Yes**	**Total**	*p*	Odd ratio (95% CI)
**ROTEM *at enrollment***					
Non-hypercoagulable	23 (82%)	5 (18%)	28	*0.011*	5.65 (1.4-22.6)
Hypercoagulable	7 (44%)	9 (56%)	16		
Total	30 (68%)	14 (32%)	44		
**ROTEM *at discharge***					
Non-hypercoagulable	23 (82%)	5 (18%)	28	*0.003*	7.66 (1.9-31.1)
Hypercoagulable	6 (38%)	10 (62%)	16		
Total	29 (66%)	15 (34%)	44		
**ROTEM *one month after discharge***					
Non-hypercoagulable	28 (76%)	9 (24%)	37	*0.002*	18.66 (2-176.4)
Hypercoagulable	1 (14%)	6 (86%)	7		
Total	29 (66%)	15 (34%)	44		

CI, confidence interval

Supplementary Table S6: Link between symptoms at three months after discharge and previous or synchronous ROTEM profile (non-hypercoagulable=normal/hemorrhagic).

	**Symptoms at three months after discharge**				
	**No**	**Yes**	**Total**	*P*	*Odd ratio (95% CI)*
**ROTEM *at enrollment***					
Non-hypercoagulable	25(89%)	3(11%)	28	*0.01*	6.22 (1.3-29.5)
Hypercoagulable	9(56%)	7(44%)	16		
Total	34(77%)	10(23%)	44		
**ROTEM *at discharge***					
Non-hypercoagulable	23(82%)	5(18%)	28	*0.30*	2.09 (0.5-8.8)
Hypercoagulable	6(38%)	10(62%)	16		
Total	29(66%)	15(34%)	44		
**ROTEM *one month after discharge***					
Non-hypercoagulable	32(86%)	5(14%)	37	*< 0.01*	15 (2.3-99.6)
Hypercoagulable	2(29%)	5(71%)	7		
Total	34(77%)	10(23%)	44		
**ROTEM *three months after discharge***					
Non-hypercoagulable	33(80%)	8(20%)	41		
Hypercoagulable	1(33%)	2(67%)	3	*0.05*	8.25 (0.7-102.7)
Total	34(77%)	10(23%)	44		

CI,confidence interval

Supplementary Table S7: Link between the presence of symptoms at one month after discharge and previous coagulation test or ROTEM parameter values. Quantitative variables are depicted as mean ± standard error of the mean (SEM) and they were analysed by Student's t-test, if they were normally distributed. Quantitative variables are depicted as median (inter-quartile range - IQR) and they were analysed by Mann-Whitney U-test if they were not normally distributed. P-values <0.05 were considered significant. aPTT, activated partial thromboplastin time; INR, international normalised ratio; MPV, mean platelet volume; CT, clotting time; CFT, Clot formation time, MCF, Maximum clot firmness; A10 and A20, Amplitudes at 10 and 20 minutes respectively; Alp, α-angle; LI30 and LI60, the percentage of thrombus lysis in 30 and 60 minutes respectively.

	**Symptomatic at 1 month post-discharge**		
	Yes (N=16)	No (N=28)	
**Values at enrolment**			p
Platelets, x 10^3/μL	274 (181-328)	179 (162-288)	0.09
aPTT, sec	30.9 (29.5-38.3)	32 (28.1-36.6)	0.68
D_Dimers, μg/mL	0.65 (0.47-1.41)	0.76 (0.43-1.19)	0.68
INR	1.03 (0.97-1.12)	1.08 (1-1.19)	0.11
MPV, fL	10.7 (10.2-11.2)	10.3 (9.9-11.1)	0.26
A10, mm	44.3 ±3.2	42.4 ±1.8	0.58
A20, mm	55.9 ±2.6	53.6 ±1.4	0.41
Alp, degrees	53.5 ±3.5	50.9 ±2.1	0.5
CFT, sec	176.5 (126-294)	258.5 (157-304)	0.46
CT, sec	591 (418-778)	578.5 (488-684)	0.79
Li30, %[#]	100 (100-100)	100 (100-100)	0.9
Li60, %	94 ±1.07	92.1 ±0.87	0.18
MCF, sec	60 ±2.17	56.9 ±1.35	0.2
**Values at discharge**			
Platelets, x10^3 /μL	427 (274-542)	266.5 (197-403)	**0.03**
aPTT, sec	29.5 (26.5-32.8)	31.7 (28.6-34.9)	0.08
D_Dimers, μg/mL	0.51 (0.34-1.01)	0.54 (0.42-0.95)	0.76
INR	1.08 (1.01-1.18)	1.07 (1.04-1.12)	0.78
MPV, fL	10.2 (10.2-10.8)	10.6 (9.8-11.1)	0.72
A10, mm	54 (45-56)	42 (36-47)	**<0.01**
A20, mm	63 (54-64)	53 (46-58)	**<0.01**
Alp, degrees	57.5 ±2.47	49.5 ±1.67	**<0.01**
CFT, sec	183.5 (125-241)	207.5 (183-289)	0.09
CT, sec	519 ± 23.7	564 ± 22.6	0.2
Li30, %	100 (100-100)	100 (100-100)	0.26

Li60, %	94 (91-98)	95 (93-98)	0.27
MCF, sec	63 ±1.71	57 ±1.46	**0.02**

Supplementary Table S8: Link between the presence of symptoms at three months after discharge and previous coagulation test or ROTEM parameter values. Quantitative variables are depicted as mean ± standard error of the mean (SEM) and they were analysed by Student's t-test, if they were normally distributed. Quantitative variables are depicted as median (inter-quartile range - IQR) and they were analysed by Mann-Whitney U-test if they were not normally distributed. P-values <0.05 were considered significant. aPTT, activated partial thromboplastin time; INR, international normalised ratio; MPV, mean platelet volume; CT, clotting time; CFT, Clot formation time; MCF, Maximum clot firmness; A10 and A20, Amplitudes at 10 and 20 minutes respectively; Alp, α-angle; LI30 and LI60, the percentage of thrombus lysis in 30 and 60 minutes respectively.

	**Symptomatic at 3 months post-discharge**		
	Yes (N=10)	No (N=34)	
**Values at enrolment**			p
Platelets, x10^3 /μL	239 (152-296)	193 (166-290)	0.99
aPTT, sec	32.2 (28.7-35.8)	31.8 (29.5-37)	0.93
D-Dimers, μg/mL	0.85 (0.47-1.38)	0.66 (0.42-1.23)	0.39
INR	1.08 (0.98-1.17)	1.07 (1-1.17)	0.98
MPV, fL	11.1 (10.4-11.6)	10.3 (10-10.9)	0.06
A10, mm	44.6 ± 4.3	42.6 ± 1.7	0.62
A20, mm	55.8 ± 3.5	54 ± 1.4	0.58
Alp, degrees	54.1 ± 4.25	51.2 ± 2	0.5
CFT, sec	172 (172-325)	258.5 (148-296)	0.64
CT, sec	577.8 ± 57.6	631 ± 32.4	0.43
Li30, %	100 (100-100)	100 (100-100)	0.99
Li60, %	94.3 ± 1.4	92.3 ± 0.8	0.24
MCF, sec	58.6 ± 3.3	57.8 ± 1.2	0.78
**Values at discharge**			
Platelets, x10^3 /μL	321 (227-526)	310 (226-449)	0.89
aPTT, sec	30 ±1	31.5 ± 0.6	0.28
D-Dimers, μg/mL	0.56 (0.45-0.95)	0.51 (0.35-1.04)	0.56
INR	1.09 (1.04-1.16)	1.06 (1.04-1.13)	0.43
MPV, fL	10.4 ± 0.18	10.5 ± 0.16	0.59
A10, mm	47.5 ± 2.9	44.1 ± 1.6	0.31
A20, mm	59 ± 2.6	54.8 ±1.4	0.16
Alp, degrees	54 ± 2.9	51.9 ±1.7	0.57
CFT, sec	203.5 (171-236)	198 (165-281)	0.98
CT, sec	568.5 (488-626)	543.5 (461-597)	0.57

Li30, %	100 (100-100)	100 (100-100)	0.91
Li60, %	95.7 ± 0.9	94.4 ± 0.7	0.34
MCF, sec	61.2 ± 2.4	58.3 ± 1.3	0.3
**Values at 1 month after discharge**			
Platelets, x10^3 /µL	250.6 ± 22.8)	260 ±10.9	0.69
aPTT, sec	31.6 (29-38)	31.2 (30.3-34.3)	0.92
D-Dimers, µg/mL	0.27 (0.21-1)	0.3 (0.24-0.5)	0.85
INR	1 ± 0.02	0.98 ± 0.01	0.56
MPV, fL	10.6 ± 0.16	10.5 ± 0.1	0.44
A10, mm	49.6 ± 2.3	40.5 ± 1.3	**<0.01**
A20, mm	58.5 ± 1.7	50.3 ± 1.2	**<0.01**
Alp, degrees	60.9 ± 2.6	49.9 ± 1.4	**<0.001**
CFT, sec	145.5 (129-176)	236.5 (191-323)	**<0.001**
CT, sec	523 (431-641)	610.5 (520-687)	0.15
Li30, %	100 (100-100)	100 (100-100)	1
Li60, %	96.5 (93-98)	96.5 (94-98)	0.99
MCF, sec	62.5 (55-65)	55.5 (51-58)	**<0.01**

Supplementary Table S9: Link between the lung diffusion capacity (DLco) determined at three months after discharge and previous coagulation test or ROTEM parameter values. Quantitative variables are depicted as mean ± standard error of the mean (SEM) and they were analysed by Student's t-test, if they were normally distributed. Quantitative variables are depicted as median (inter-quartile range - IQR) and they were analysed by Mann-Whitney U-test if they were not normally distributed. Normal DLco values are those > 80% of the predicted value. aPTT, activated partial thromboplastin time; INR, international normalised ratio; MPV, mean platelet volume; CT, clotting time; CFT, Clot formation time, MCF, Maximum clot firmness; A10 and A20, Amplitudes at 10 and 20 minutes respectively; Alp, α-angle; LI30 and LI60, the percentage of thrombus lysis in 30 and 60 minutes respectively.

	**DLco Normal** (N=22)	**DLco Decreased** (N=22)	P
**Values at enrolment**			
Platelets, x10^3 /µL	175 (157-274.5)	280 (175-312)	**0.03**
aPTT, sec	32 (29.1-37.4)	30.9 (28.7-35)	0.45
D-Dimers, µg/mL	0.58 (0.42-1.21)	0.8 (0.47-1.34)	0.3
INR	1.05 (1-1.18)	1.09 (1-1.16)	0.7
MPV, fL	10.4 (9.9-11)	10.6 (10.2-11.3)	0.17
A10, mm	40.4 ± 2	45.7 ± 2.4	0.09
A20, mm	51.8 ± 1.6	57.1 ± 1.9	**0.04**
Alp, degrees	48.6 ± 2.3	55.1 ± 2.7	0.07
CFT, sec	263.5 (184-333)	171.5 (128-279)	0.09
CT, sec	631 (496-863)	533 (454-704)	0.21
Li30, %	100 (100-100)	100 (100-100)	0.48
Li60, %	91.9 ± 1.1	93.6 ± 0.8	0.24
MCF, sec	55 ± 1.43	61 ± 1.7	**<0.01**
**Values at discharge**			
Platelets, x10^3 /µL	256.5 (183-426)	515 (260-657)	**0.04**
aPTT, sec	32.1 ± 0.85	30.2 ± 0.65	0.09
D-Dimers, µg/mL	0.48 (0.32-0.8)	0.55 (0.45-1.06)	0.16
INR	1.05 (1.03-1.11)	1.08 (1.04-1.16)	0.56
MPV, fL	10.5 ± 0.2	10.5 ± 0.16	0.99
A10, mm	41.5 (34.7-46)	52 (42.2-56)	**<0.001**
A20, mm	51.4 ±1.4	60.2 ±1.6	**<0.001**
Alp, degrees	48 ±1.8	56.8 ± 2	**<0.01**
CFT, sec	214 (186-303)	183.5 (127-242)	**0.02**
CT, sec	559.2 ± 27.7	536.7 ±19.9	0.51
Li30, %	100 (100-100)	100 (100-100)	0.99
Li60, %	94.6 ± 0.81	94.9 ± 0.76	0.81
MCF, sec	55.4 ± 1.57	62.4 ± 1.45	**<0.01**
**Values at one month after discharge**			
Platelets, x10^3 /µL	240 ±12.3	276 ± 14.5	0.07
aPTT, sec	31.1 (30.2-36.1)	32.3 (29.6-34.1)	0.87
D-Dimers, µg/mL	0.34 (0.21-0.56)	0.31 (0.24-0.5)	0.7
INR	0.98 ± 0.01)	0.99 ± 0.01	0.64
MPV, fL	10.4 ± 0.13	10.6 ± 0.11	0.3
A10, mm	41 (38-45.5)	43.5 (40-52.5)	0.06
A20, mm	51.5 (46-54)	54 (50-61)	**0.01**
Alp, degrees	50.5 (44-56)	54.5 (48-63)	**0.04**
CFT, sec	248 (189-362)	198 (144-246)	**<0.01**
CT, sec	613.5 (523-687)	561.5 (485-654)	0.21
Li30, %	100 (100-100)	100 (100-100)	0.99
Li60, %	96.7 ± 0.6)	95.2 ± 0.7	0.1
MCF, sec	55 (52-57.5)	57.5 (54-64)	**0.04**

Supplementary Table S10: Link between forced vital capacity (FVC % of the predicted value) determined three months after discharge and the previous or synchronous ROTEM profile (non-hypercoagulable=normal/hemorrhagic). FVC > 80% of the predicted value is considered normal. Comparisons were made using Fisher's exact test.

	FVC (% of the predicted value)				
	**Normal**	**Decreased**	**Total**	*p*	*OR (95% CI)*
**ROTEM *At Enrollment***					
Non-hypercoagulable	27	1	28	>0.99	0.82 (0.05-7.52)
Hypercoagulable	16	0	16		
Total	43	1	44		
**ROTEM *At Discharge***					
Non-hypercoagulable	27	1	28		
Hypercoagulable	16	0	16	>0.99	0.82 (0.05-7.52)
Total	43	1	44		
**ROTEM *one month after discharge***					
Non-hypercoagulable	36	1	37		
Hypercoagulable	7	0	7	0.47	2.31 (0.14-21.33)
Total	43	1	44		

Supplementary Table S11: Link between lung diffusion capacity/alveolar ventilation (DLco/VA % of the predicted value) determined three months after discharge and the previous or synchronous ROTEM profile (non-hypercoagulable=normal/hemorrhagic). DLco/VA>80% of the predicted value is considered normal. Comparisons were made using Fisher's exact test.

	DLco/VA (% of the predicted value)				
	**Normal**	**Decreased**	**Total**	*p*	*OR (95% CI)*
**ROTEM*At Enrollment***					
Non-hypercoagulable	26	2	28	0.99	0.87 (0.06-7.96)
Hypercoagulable	15	1	16		
Total	41	3	44		
**ROTEM *At Discharge***					
Non-hypercoagulable	26	2	28		
Hypercoagulable	15	1	16	0.99	0.87 (0.06-7.96)
Total	41	3	44		
**ROTEM *one month after discharge***					
Non-hypercoagulable	34	3	37		
Hypercoagulable	7	0	7	0.99	1.09 (0.08-8.75)
Total	41	3	44		

Supplementary Table S12: Link between alveolar ventilation (VA, % of predicted value) determined three months after discharge and the previous or synchronous ROTEM profile (non-hypercoagulable=normal/hemorrhagic). Comparisons were made using Fisher's exact test.

	VA (% of predicted value)				
	**Normal**	**Decreased**	**Total**	*p*	*OR (95% CI)*
**ROTEM*At Enrollment***				0.11	3.2 (0.93-10.44)
Non-hypercoagulable	20	8	28		
Hypercoagulable	7	9	16		
Total	27	17	44		
**ROTEM *At Discharge***					
Non-hypercoagulable	20	8	28	0.11	3.2 (0.93-10.44)
Hypercoagulable	7	9	16		
Total	27	17	44		
**ROTEM *one month after discharge***					
Non-hypercoagulable	25	12	37	0.09	5.21 (0.84-27.98)
Hypercoagulable	2	5	7		
Total	27	17	44		
